# Antibiotic Stewardship: Comparing Meropenem De-Escalation and Continued Therapy When Cultures Show No Multi-Drug Resistance

**DOI:** 10.7759/cureus.106517

**Published:** 2026-04-06

**Authors:** Sean Swieczkowski, Ivan A Hernandez, Edwina E Brathwaite, Victoria Y McCue, Maricar Castillo, Anshul Saxena

**Affiliations:** 1 Clinical Pharmacy, Baptist Health South Miami Hospital, Miami, USA; 2 Pharmacy, Baptist Health South Florida, Miami, USA; 3 Nursing, Baptist Health Doctors Hospital, Miami, USA; 4 Biostatistics, Miami Cardiac and Vascular Institute, Baptist Health South Florida, Miami, USA

**Keywords:** antibiotic resistance (abr), antibiotic stewardship program, carbapenem use, id critical care, meropenem utilization

## Abstract

Background: Current practice recommends de-escalation of antibiotics once cultures are available and show susceptibility to less broad-spectrum antibiotics. Some providers still choose not to follow cultures and continue broad-spectrum antibiotics. This can lead to harm to the patient and resistance to antibiotics in the patient and in the community.

Methods: This is a retrospective cohort study conducted via chart review utilizing electronic healthcare record reports. Patients were screened for inclusion if they were admitted into the Intensive Care Unit and received meropenem for longer than 48 hours from January 1st, 2021, to December 31st, 2023. A total of 118 patients were included. The primary outcome was an increase in mortality in patients receiving meropenem when cultures showed no multidrug resistance (Extended Spectrum Beta-Lactamase, i.e., ESBL) and was not de-escalated, versus when cultures showed no multidrug resistance (ESBL) and was de-escalated. The secondary outcome was comparing the length of stay and duration of antibiotics in the meropenem group versus patients who were de-escalated from meropenem, as well as the cost of meropenem and the cost to the hospital.

Results: No statistically significant difference in mortality was observed (p = 0.182), although a numerical trend toward lower mortality in the de-escalation group was noted. The median length of stay was 14 days in the non-de-escalated group and 11 days in the de-escalated group (p = 0.439), indicating no statistical significance. The median days of therapy in the non-de-escalated group were seven days, and in the de-escalated group were four days (p < 0.001), showing statistical significance. The median meropenem cost for the non-de-escalated group was $2,286, while for the de-escalated group it was $1,344 (p = 0.003), showing a significant difference. The median hospital cost for the non-de-escalated group was $402,002.50, while in the de-escalated group, it was $200,334.50 (p = 0.5), showing no statistical significance.

Conclusion: In hospitalized critical care patients, the de-escalation of meropenem when cultures indicate to do so can not only potentially decrease mortality but also decrease length of stay. Further studies could provide more robust data to support our findings and challenge providers who choose to use broad-spectrum antibiotics even when cultures indicate de-escalation.

## Introduction

Antibiotic stewardship refers to the coordinated efforts to optimize the prescribing and use of antimicrobial agents to improve patient outcomes and reduce adverse effects [1]. Its overarching goal is to ensure continuous quality improvement as clinical guidelines and evidence evolve. In February 2020, the American Thoracic Society, together with the American Association of Critical-Care Nurses, the American College of Chest Physicians, the Centers for Disease Control and Prevention, and the Society of Critical Care Medicine, issued a statement on the importance of antibiotic stewardship in the intensive care unit (ICU). This statement highlighted that the ICU is a setting characterized by high utilization of broad-spectrum antimicrobials, driven in part by critical care physicians' concerns regarding inadequate pathogen coverage and the perceived need for escalation [2]. The authors emphasized the need to consider both the potential adverse effects of antimicrobial therapy and the evidence demonstrating the safety and benefits of stewardship practices [3].

Meropenem is a broad-spectrum carbapenem antibiotic with activity against *Pseudomonas aeruginosa*, *Acinetobacter baumannii*, *Klebsiella pneumoniae*, extended-spectrum β-lactamase-producing gram-negative organisms (ESBL), *Streptococcus* species, methicillin-susceptible *Staphylococcus aureus*, and *Enterococcus faecalis* [4,5]. It is administered intravenously at doses of 1-2 g every eight hours in patients with normal renal function [6,7]. Meropenem is commonly used in critically ill patients with infections, including sepsis, bacterial meningitis, pneumonia, intra-abdominal infections, and urinary tract infections [6]. Within our institution, meropenem is commonly utilized in patients with multi-drug resistant (MDR) organisms, such as those that are ESBL-producing bacteria [8].

Primary objective

Determine whether critically ill patients receiving meropenem experience different rates of all-cause in-hospital mortality when culture results support de-escalation and de-escalation is implemented.

Secondary objective

Compare the length of stay, duration of antimicrobial therapy, hospital cost, and meropenem cost between patients who remained on meropenem and those whose therapy was de-escalated.

By categorizing patients into these two groups, this study aims to evaluate the safety and impact of antimicrobial stewardship practices within the critically ill population.

## Materials and methods

This retrospective cohort study of patient records was approved by the Institutional Review Board (IRB). The study evaluated critically ill patients admitted to a hospital in the South Florida region who received meropenem during their hospitalization. The primary objective was to determine whether all-cause in-hospital mortality differed between patients who received meropenem with no multidrug-resistant (MDR) organisms and those who were de-escalated based on culture results. Secondary objectives included comparisons of hospital length of stay (LOS), duration of antibiotic therapy (DOT), Meropenem cost, and hospital cost.

A total of 118 electronic health records from January 1, 2021, to December 31, 2023, were reviewed. Of these, 105 (89%) patients continued meropenem therapy, while 13 (11%) patients underwent de-escalation.

Inclusion criteria included patients admitted into the ICU who received meropenem for longer than 48 hours. Exclusion criteria included being younger than 18 years old, being pregnant, being a prisoner, having a hospitalization LOS less than 72 hours, or having a documented allergy to Piperacillin/Tazobactam and/or Cefepime. De-escalation was defined as cases in which culture results were negative, and no multidrug-resistant organisms were identified. The absence of multidrug resistance was determined by the lack of ESBL-producing organisms, as evidenced by cultures demonstrating susceptibility to Ceftriaxone. Patient data was collected using the ProDiver analytical tool based on the criteria outlined above.

Collected data included demographics, comorbidities, infection source, microbiological data, antibiotic use, LOS, DOT, and hospital costs. Statistical analyses included chi-squared or Fisher’s exact tests for categorical variables and Kruskal-Wallis or t-tests for continuous variables, as appropriate. A Cox proportional hazards model was used to assess the impact of de-escalation on mortality, adjusting for potential confounders.

## Results

The baseline patient demographics and characteristics for all 118 patients included in this study are outlined in Table 1.

**Table 1:** Baseline Patient Characteristics N/A: Not applicable

Variable	MDR No De-escalation with Mortality	MDR No De-escalation without Mortality	MDR De-escalation with Mortality	MDR De-escalation without Mortality	No MDR No De-escalation with Mortality	No MDR No De-escalation without Mortality	No MDR De-escalation with Mortality	No MDR De-escalation without Mortality
Number of patients (%)	8 (6.8%)	40 (34%)	0 (0%)	5 (4.5%)	23 (19%)	34 (29%)	1 (0.8%)	7 (5.9%)
Age, median [Q1, Q3]	85.5 [73.75-88.01]	80 [72.01-86.01]	N/A	81 [80.01-82.01]	74 [68.5-79.01]	76 [59.75-83.25]	89	80 [75.01-82.5]
COVID Positive, n (% male)	25	7.5	N/A	20	39.1	6	100	0
Gender, n (% male)	25	42.5		20	56.51	41	100	57.14
Renal, n (%)	12.5	57.5	N/A	80	34.78	41	100	57.14
Cardiac, n (%)	50	57.5	N/A	100	60.87	62	100	71.43
Respiratory, n (%)	25	22.5	N/A	40	26.09	44	100	28.57
PSI at start of Meropenem, median [Q1, Q3]	131 [119.25, 160.25]	130.5 [114.01, 168.01]	N/A	159 [152.01, 198.01]	153 [132.01, 187.5]	134.5 [86.75, 161.01]	149	141 [126.5, 154.01]

Patients with MDR organisms

Among patients with MDR organisms who did not undergo de-escalation, mortality occurred in eight patients (6.8%). These patients were elderly and predominantly female. COVID-19 co-infection was present in 25% of patients. Cardiac dysfunction was the most common comorbidity, followed by respiratory and renal dysfunction. The median Pneumonia Severity Index (PSI) at initiation of meropenem was 131 (IQR 119.25-160.25).

In contrast, 40 patients (34%) with MDR organisms survived without de-escalation. This group was slightly younger, with a comparable median PSI of 130.5 (IQR 114.01-168.01). Comorbidity patterns were similar overall, though renal dysfunction was more frequently observed in survivors. Among the five patients (4.5%) with MDR organisms who underwent de-escalation, no mortality was observed despite a higher median PSI of 159 (IQR 152.01-198.01).

Patients with non-MDR organisms

Among patients without MDR organisms who did not undergo de-escalation, 23 (19%) died. The median PSI score was 153 (IQR 132.01-187.5) with a notable prevalence of COVID-19 co-infection and cardiac dysfunction.

Thirty-four patients (29%) without MDR organisms survived without de-escalation. Their median PSI was lower at 134.5 (IQR 86.75-161.01), with similar distributions of comorbid conditions.

In the de-escalation subgroup without MDR organisms, one patient (0.8%) died. Seven additional patients (5.9%) survived following de-escalation with a median PSI in this subgroup of 141 (IQR 126.5-154.01).

Primary outcome: in-hospital mortality

**Figure 1:**
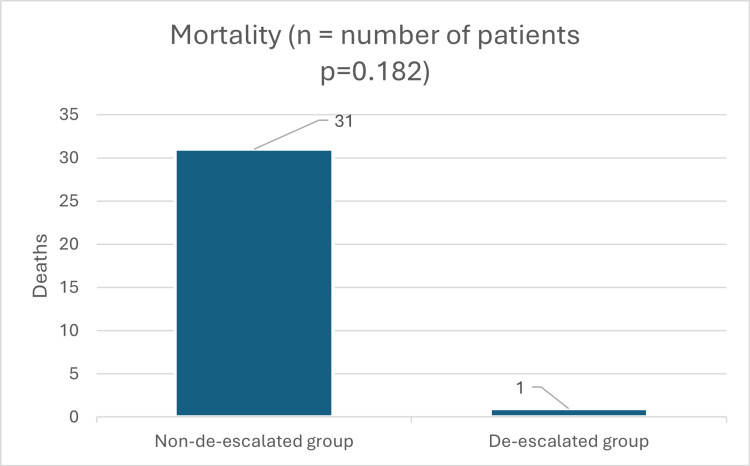
Primary Endpoint In-Hospital Mortality

Overall, in-hospital mortality was 27.1%. Mortality occurred in 29.5% of patients in the non-de-escalated group compared with 7.7% in the de-escalated group. Although mortality was numerically lower with de-escalation, this difference did not reach statistical significance (p = 0.182) (Figure 1).

Secondary outcomes 

**Figure 2:**
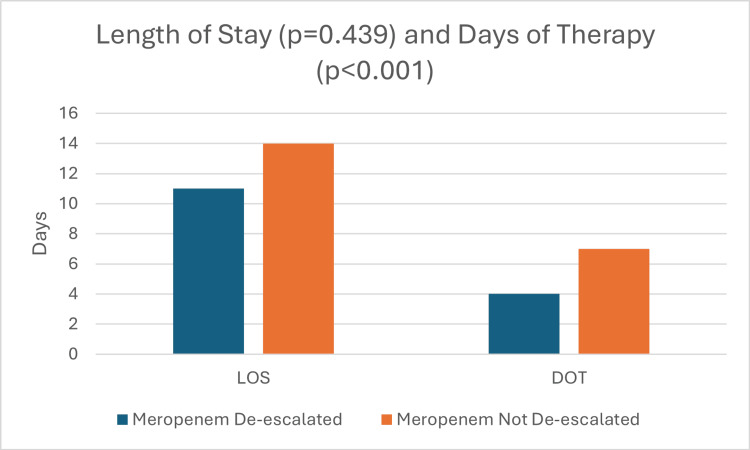
Median Length of Stay and Median Days of Therapy LOS: Length of stay, DOT: Days of therapy

Median LOS was 14 days (Interquartile range [IQR]: 9.01-22.01) in the non-de-escalation group and 11 days (IQR: 8.01-25.01) in the de-escalated group (p = 0.439) (Figure 2). Median DOT was four days (IQR: 4.01-5.01) in the de-escalated group and seven days (IQR: 6.01-9.01) in the non-de-escalation group (p < 0.001) (Figure 2).

**Figure 3:**
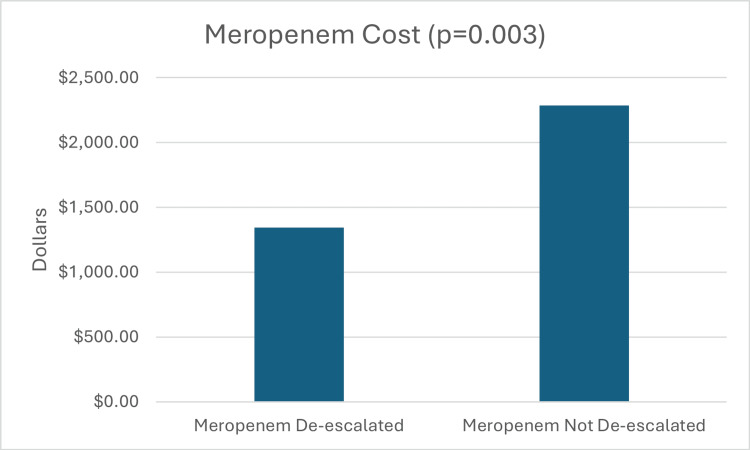
Secondary Outcome: Meropenem Cost A) Meropenem de-escalated group had a median meropenem cost of $1,344. B) Meropenem not de-escalated group had a median meropenem cost of $2,286

**Figure 4:**
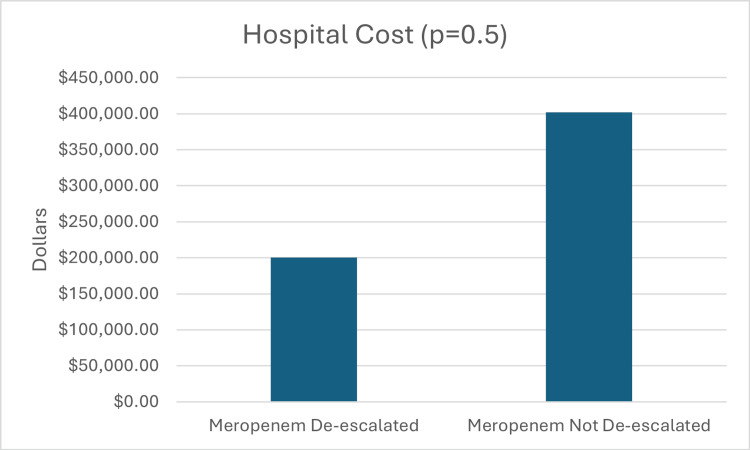
Secondary Outcome: Hospital Cost A) Meropenem de-escalated group had a hospital cost of $200,334.50. B) Meropenem not de-escalated group had a hospital cost of $402,002.50.

The median meropenem cost was $1,344 in the de-escalated group, while the median meropenem cost in the non-de-escalated group was $2,286. This result was statistically significant (p = 0.003) (Figure 3). The median hospital cost was $200,334.50 in the de-escalated group, while the non-de-escalated median hospital cost was $402,002.50. There was no significant difference in total hospital cost between groups (p = 0.500) (Figure 4).

**Figure 5:**
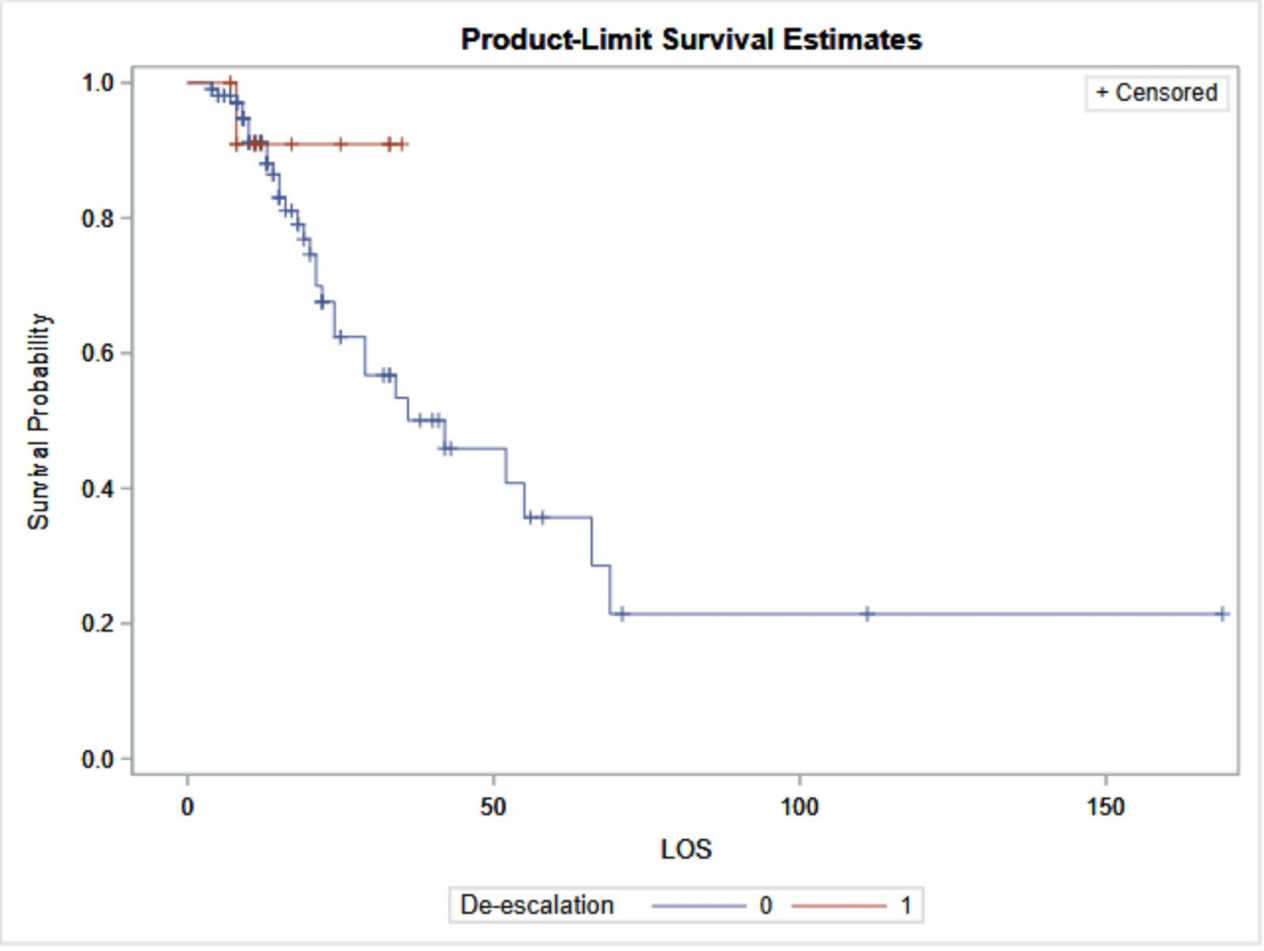
Multivariable Analysis A) LOS: Length of stay. B) Solid blue line: Not de-escalated (0) C) Solid red line: de-escalated (1)

Figure 5 shows the survival probability based on whether the antibiotic was de-escalated or not. The red line (1) describes the patient population in the de-escalation group, and the blue line (0) describes the patient population in the non-de-escalation group. A Cox regression model adjusting for age, gender, COVID-19 status, number of comorbidities, and Pneumonia Severity Index (PSI) showed that de-escalation was not significantly associated with mortality (HR = 0.397, 95% CI: 0.052-3.036, p = 0.373).

## Discussion

There has been considerable debate regarding the optimal timing for antibiotic de-escalation. Current best practice recommends de-escalating therapy once culture results are available. If cultures remain negative, guidelines advise de-escalation; if cultures are positive, therapy should be adjusted to specifically target the identified organism. Previous positive cultures may also be used to guide initial empirical therapy [9,10].

A recent study reviewed antibiotic de-escalation in critically ill patients with negative clinical cultures and found that appropriate de-escalation resulted in a shorter length of stay (p< 0.001), lower incidence of acute kidney injury (AKI) (p = 0.031), and found no difference in all-cause ICU mortality (p =0.227) [11,12]. In this review, several cases were identified in which meropenem was continued despite negative culture results.

This study specifically examined a subset of patients with MDR-producing organisms whose bacterial isolates remained susceptible to carbapenem antibiotics, as well as patients with less extensively drug-resistant pathogens who met criteria for antimicrobial de-escalation. No carbapenem-resistant organisms were identified among the cultures analyzed in this study population. The findings of this study are most applicable to clinical settings involving ESBL-producing organisms and may not be generalizable to cases involving highly resistant bacteria such as carbapenem-resistant *Enterobacterales *(CRE).

The primary outcome of this study was mortality. Mortality in the non-de-escalated group was 29.5% (31 of 105 patients), compared with 7.7% (1 of 13 patients) in the de-escalated group. Although the p-value of 0.182 indicates no statistical significance, this may be attributable to the small sample size. In Figure 5, we see the survival probability, which was not statistically significant. Even though this is not statistically significant, the graph does show some clinical significance. The de-escalated group shows a shorter LOS and a lower mortality rate than the non-de-escalated group. The data suggest a potential trend toward reduced mortality with carbapenem de-escalation; however, this finding did not reach statistical significance, precluding definitive conclusions. A larger study population may provide sufficient power to detect a statistically significant effect.

Secondary outcomes demonstrated similar patterns. The median LOS was 14 days in the non-de-escalated group and 11 days in the de-escalated group (p = 0.439), which was not statistically significant but suggests a trend toward shorter hospitalizations with de-escalation. DOT was significantly reduced in the de-escalated group (four days) compared with the non-de-escalated group (seven days) (p < 0.001). Meropenem costs were also significantly lower in the de-escalated group ($1,344) compared with the non-de-escalated group ($2,286) (p = 0.003). However, there was no significant difference in total hospital costs between the groups (p = 0.5). Although statistical significance was reached for DOT and meropenem costs, further studies will need to be done to confirm outcomes based on the small sample size.

Baseline characteristics, including comorbidities and Pneumonia Severity Index (PSI) scores [13-15], were balanced between groups through randomization to minimize confounding. All patients were admitted to the intensive care unit (ICU) to ensure consistency in the level of care across the study population.

This study has several limitations. The small sample size reduces statistical power and limits the generalizability of the findings. Selection bias may also be present, including attrition bias, as some patients were discharged and not continued to be followed. Additionally, all patients were treated in the intensive care unit, where mortality is influenced by multiple clinical factors. For instance, outcomes may differ significantly between patients treated for a urinary tract infection and those with septic pneumonia, making it difficult to attribute mortality solely to antimicrobial use.

There were also imbalances between study groups, with a larger non-de-escalated cohort compared to the de-escalated group, which may affect the statistical validity of the results. Finally, the retrospective, observational design further limits the study, as the lack of randomization introduces the potential for confounding variables.

## Conclusions

De-escalation of meropenem therapy guided by culture and susceptibility results appears to be comparably effective to prolonged broad-spectrum treatment and may offer additional advantages in terms of patient safety and overall cost-effectiveness. By limiting unnecessary exposure to meropenem, de-escalation strategies have the potential to reduce adverse drug events, antimicrobial resistance, and healthcare expenditures without compromising clinical outcomes. The primary outcome of this study found no statistically significant difference in in-hospital mortality between patients who were de-escalated from meropenem versus patients who were not de-escalated from meropenem. Statistical significance was achieved in the DOT and meropenem cost; no statistical significance in the LOS and hospital cost was found. There might have been positive trends in mortality and LOS, but since there was no statistical significance, we cannot conclude the impact this study may have. These findings highlight the need for larger, adequately powered studies to further evaluate the impact of meropenem de-escalation and to determine whether these observed benefits reach statistical significance in broader patient populations.
